# Ocean submesoscales as a key component of the global heat budget

**DOI:** 10.1038/s41467-018-02983-w

**Published:** 2018-02-22

**Authors:** Zhan Su, Jinbo Wang, Patrice Klein, Andrew F. Thompson, Dimitris Menemenlis

**Affiliations:** 1 Jet Propulsion Laboratory, California Institute of Technology, Pasadena, CA 91109 USA; 2 Environmental Science and Engineering, California Institute of Technology, Pasadena, CA 91125 USA; 3 Laboratoire d’Océanographie Physique et Spatiale, Brest, 29200 France

## Abstract

Recent studies highlight that oceanic motions associated with horizontal scales smaller than 50 km, defined here as submesoscales, lead to anomalous vertical heat fluxes from colder to warmer waters. This unique transport property is not captured in climate models that have insufficient resolution to simulate these submesoscale dynamics. Here, we use an ocean model with an unprecedented resolution that, for the first time, globally resolves submesoscale heat transport. Upper-ocean submesoscale turbulence produces a systematically-upward heat transport that is five times larger than mesoscale heat transport, with winter-time averages up to 100 W/m^2^ for mid-latitudes. Compared to a lower-resolution model, submesoscale heat transport warms the sea surface up to 0.3 °C and produces an upward annual-mean air–sea heat flux anomaly of 4–10 W/m^2^ at mid-latitudes. These results indicate that submesoscale dynamics are critical to the transport of heat between the ocean interior and the atmosphere, and are thus a key component of the Earth’s climate.

## Abstract (web summary)

Oceanic motions associated with horizontal scales smaller than 50 km remain unresolved in climate models. Here the authors show that motions in this scale range are critical to the global transport of heat between the ocean interior and the atmosphere, and are thus a key component of the Earth’s climate.

## Introduction

**Fig. 1:**
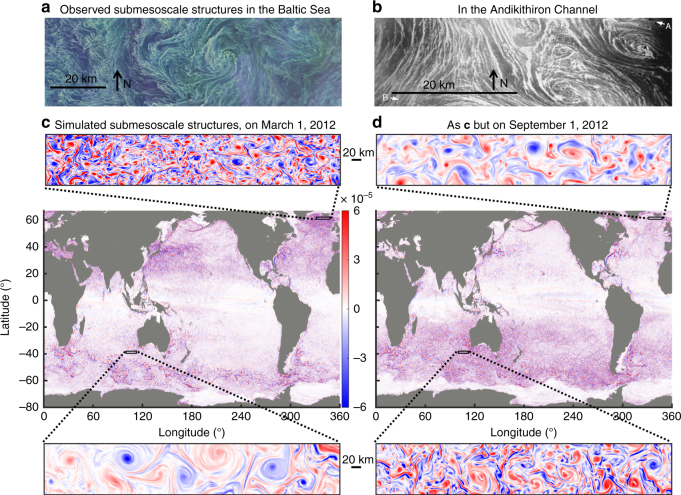
Overview of global submesoscale structures and distributions. **a** Satellite image of a large bloom of cyanobacteria in the Baltic Sea on August 11, 2015 showing submesoscale eddies, fronts, and filaments. Figure 1a sourced from NASA (images by Norman Kuring, NASA's Ocean Color Web). See https://landsat.visibleearth.nasa.gov/view.php?id=86449. **b** Observations^11^ of submesoscale structures from sunglitter in the Andikithiron Channel northwest of Crete on October 7, 1984. Ship tracks are labeled A, B. **c**, **d** Simulated global snapshot of ocean turbulence at ~2-km resolution, as well as expanded views for local regions, for **c** Northern and **d** Southern Hemisphere winters. The quantity shown is relative vorticity (s^−1^), a measure of the spin of fluid parcels, that emphasizes fast-rotating submesoscale turbulence especially in the winter hemisphere (see Supplementary Figure 1 for Rossby number). The same global maps but with a higher pixel resolution can be found at web.gps.caltech.edu/~zhan/nailed/Figure1a.png

Variations in the heat content of the ocean–atmosphere-coupled system influence Earth’s climate^1,2^. Turbulent motions in the upper ocean (down to ~300 m depths) occur across a broad range of spatial scales, from ~1000 km down to ~1 km and even smaller scales, and influence the partitioning of heat between the ocean and atmosphere as suggested by local studies^3,4^. Satellite images during the past four decades reveal broadly distributed submesoscale (~0.1–50 km) turbulence over the global ocean^5^, typically characterized by surface frontal structures in the form of eddies and elongated filaments (Fig. 1a, b). Only recently, regional numerical and observational studies^4^ have shown that submesoscales may explain more than 50% of the total vertical velocity variance in the ocean’s upper 300 m. This implies that submesoscale fluxes are a potentially efficient route for the vertical transport of heat, nutrients, oxygen, and climatically important dissolved gases. Submesoscale structures are produced by several classes of surface frontal instabilities^3–11^ that tend to be intensified in the winter when the mixed layer depth is large. Additionally, submesoscales may transport heat vertically from cold to warm waters (up-gradient)^4–12^, the opposite sense of traditional subgrid-scale parameterizations (down-gradient) in climate models. This unique property is not captured in climate models that have insufficient resolution to simulate these submesoscale dynamics. Useful parameterizations^13^ have been proposed and implemented for certain submesoscale processes, although these involve sometimes-unsettled coefficients while other submesoscale processes remain uncaptured, e.g., wind-front interactions and frontogenesis. The full impact of submesoscale turbulence on ocean vertical heat transport and air–sea heat exchange has not previously been directly quantified on a global scale, representing a potential gap in our understanding of the evolution of global oceanic and atmospheric heat content^13^. Accurately assessing the impact of submesoscale turbulence requires explicit resolution of motions at these scales, which remains computationally challenging for global ocean simulations.

In this study, we make a major advance toward realizing this challenge by using a novel global ocean simulation with an unprecedented ~2 km horizontal resolution that, for the first time, globally resolves the ocean submesoscale heat transport at the 10–50-km scale range. We find that submesoscale turbulence produces a large and systematically upward heat transport globally throughout the upper ocean: the winter-time averages are 20–100 W/m^2^ across most of the mid-latitudes, peaking at 500–1000 W/m^2^ over shorter intervals of days to weeks. These amplitudes, consistent with recent in situ observations^11^, are comparable to air–sea heat fluxes, and are more than five times larger than the mesoscale vertical heat transport in most regions of the ocean. Comparison with a lower resolution simulation shows that submesoscale vertical heat transport warms the sea surface by 0.06–0.3 °C and produces an upward annual mean air–sea heat flux anomaly of 4–10 W/m^2^ in most mid-latitude areas. The latter is comparable to climatological air–sea heat fluxes. These results suggest that submesoscale processes are critical to setting the magnitude of the vertical heat flux between the interior ocean, the surface ocean, and the atmosphere, as well as the geographic location and temporal variability of these fluxes over days to months, and are therefore a key component of the global heat budget.

## Results

### An ultrahigh-resolution global ocean simulation

Here, we use a global ocean model with unprecedented resolution (1/48°, 90 levels, see “Methods”). The horizontal resolution, ~2 km at mid-latitude, captures physical processes at wavelengths down to a scale of ~10 km^14,15^. This ocean simulation has been evaluated, locally, using in situ observations in terms of the kinetic energy levels at different wavelengths and frequencies^14,15^. The simulation is progressively spun up from coarser resolution (initially 1/6°), observationally constrained model solutions^16^, which results in a quasi-equilibrated and realistic upper ocean state above the first 500 m (see “Methods”). Throughout this manuscript, we use the term submesoscale to designate a spatial scale smaller than the mesoscale: a horizontal scale ≲0.5° in terms of longitude (~50 km at mid-latitudes), roughly below the first deformation radius in most of the global ocean^9,17–22^ (in terms of wavelengths). Our simulation can resolve the 10–50 km submesoscale range (see “Methods”); we discuss the potential impact of the still unresolved submesoscale range below.

### Overview of global submesoscale structures and distributions

The simulation shows that submesoscale turbulence populates the global upper ocean with coherent structures (10–50 km) embedded in the larger-scale ocean circulation (Fig. 1c, d and Supplementary Figure 1). Relative vorticity ζ, the curl of the velocity field, is a useful diagnostic for visualizing the ocean submesoscales. The relative vorticity (Fig. 1) on March 1, 2012 (Northern Hemisphere winter) and September 1, 2012 (Southern Hemisphere winter) reveals the geographic variability of submesoscale turbulence. Submesoscales in our simulation are everywhere characterized by a myriad of eddies as small as ~10 km intertwining with elongated and wavy filaments, as seen in the expanded panels of Fig. 1. There is a pronounced global seasonal signal in the ζ-field, characterized by larger magnitudes and smaller scales during the winter. Relative vorticity values comparable to Earth’s rotation rate (Supplementary Figure 1) are found in almost all ocean basins. This suggests^4,23^ strong and wide-spread vertical velocities in the upper ocean: in the 20°–60° mid-latitude band during the winter, about 51% and 2% of ocean area at 40 m depth has magnitudes larger than 10 m/day and 40 m/day, respectively (Supplementary Figure 2a–b). The above characteristics dominate not only in the Gulf Stream and Kuroshio, as already reported^18,20,24^, but also in a large number of unexplored regions that are critical for ocean–atmosphere exchange, including the Southern Ocean, the high-latitude North Atlantic, the broad subtropical oceans, and the Mediterranean, Black, and Arabian Seas. Note that these regions generally have deep mixed layer depths, which is an important factor for producing intense submesoscale motions.

### Temporal variabilities of submesoscale characteristics

**Fig. 2:**
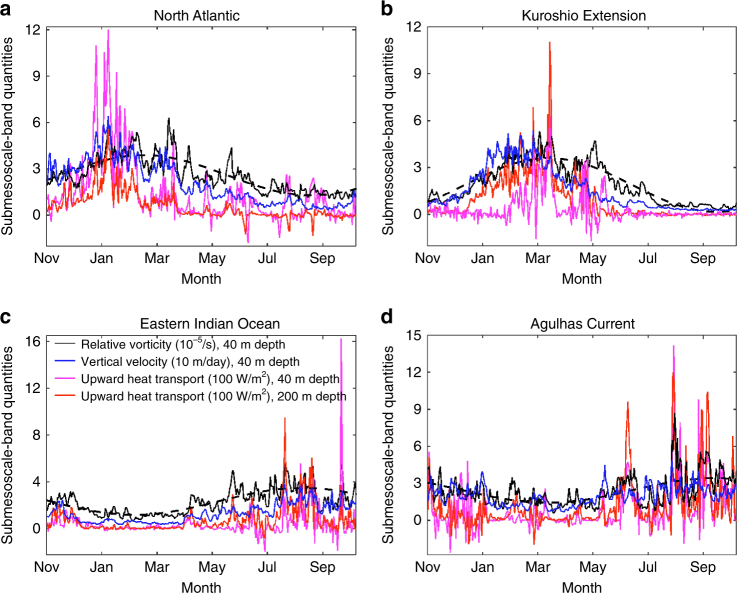
Temporal variability of submesoscale characteristics. Local time series of submesoscale band (~10–50 km, see “Methods”) root mean square (rms) relative vorticity (black), rms vertical velocity (blue), and vertical heat transport (positive: upward) at 40 m and 200 m depths (magenta and red, respectively). Dashed curves are the sinusoidal seasonal cycle fit for relative vorticity. The locations are in the **a** high-latitude North Atlantic (57°N, 26°W), **b** Kuroshio Extension (38.1°N, 156°E), **c** eastern Indian Ocean (38.3°S, 118°E), **d** Agulhas Current (40°S, 18°E). The results here are averaged over a 1° × 1° square box in each location. The correlation between the time series suggests a strong dynamic connection between the different quantities. Submesoscale upward heat transport can reach a significant amplitude up to 500–1000 W/m^2^, consistent with values inferred from recent observations^10^, and is characterized by a strong intermittency (days to weeks) and a winter-peaked seasonality

The time series of submesoscale vorticity and vertical heat transport exhibit not only a strong winter-peaked seasonality, but also a significant intermittency at daily to weekly time scales (Fig. 2, see “Methods”). The latter is associated with the short timescales of submesoscale dynamics^3,22^. The time series of the root-mean-square values of ζ and vertical velocity confirm their strong correlation (Fig. 2) and therefore the frontal character of submesoscale turbulence. This relationship is due to the ageostrophic circulation described by the omega equation^23^. The energetic vertical motions cause significant vertical heat transport at submesoscales, up to 500–1000 W/m^2^ (Fig. 2). The seasonal variability of the vertical heat flux has an amplitude of up to 200 W/m^2^, and its variability over shorter intervals of days to weeks peaks at 500–1000 W/m^2^. Our results suggest that submesoscale turbulence dramatically enhances the upper-ocean heat exchange at diverse time scales ranging from days to seasons, which applies broadly across the global ocean.

### Global patterns of submesoscale vertical heat transport

**Fig. 3:**
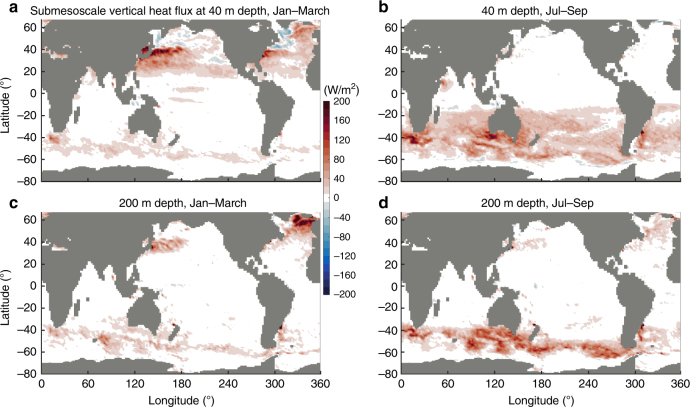
Global patterns of submesoscale vertical heat transport. Values are spatially smoothed over 3° × 3° square boxes; positive values indicate upward. The heat flux is calculated at 40 m depth (**a**, **b**) and 200 m depth (**c**, **d**) during the winter or summer seasons: **a**, **c** Jan–March mean and **b**, **d** Jul–Sep mean. In most area of mid-latitudes, vertical heat transport at submesoscales is ~20–100 W/m^2^ and is systematically upwards

The enhancement of the near-surface vertical heat transport by submesoscales is ubiquitous over the global ocean (Fig. 3). This heat transport is systematically upward (upgradient): it acts to produce a surface warming and a deeper cooling, intensified in winter. In the wintertime Southern Hemisphere, large submesoscale heat fluxes at 40-m depth cover most of the region between 20°S and 60°S (Fig. 3b), where 66% and 5% of ocean area has amplitudes larger than 20 W/m^2^ and 100 W/m^2^, respectively. At 200-m depth, submesoscale fluxes retain their large amplitudes and mostly cover the 35°–60°S latitude band (Fig. 3d), where 48% and 10% of ocean area here has amplitudes larger than 20 W/m^2^ and 100 W/m^2^, respectively. Large submesoscale heat fluxes also occur in the wintertime Northern Hemisphere (Fig. 3a, c). These are strongest in the 20°–40°N band, where 65% and 11% of ocean area has amplitudes larger than 20 W/m^2^ and 100 W/m^2^, respectively, at 40 m depth. Similar amplitudes also occur in the Atlantic subpolar and polar gyres (40°–60°N), where 61% and 19% of ocean area has amplitudes larger than 20 W/m^2^ and 100 W/m^2^, respectively, at 200 m depth. During the summer, submesoscale fluxes are still positive but have a smaller amplitude and more limited spatial coverage. Only the region between 40°–60°S, as well as 40°–60°N in the Atlantic, still have significant submesoscale fluxes, where 32% and 46% of the area in these respective latitude bands have amplitudes larger than 10 W/m^2^ at 200 m depth (Fig. 3c, d).

Importantly, in most regions the amplitudes of the submesoscale heat transport, 20–100 W/m^2^ over the winter hemisphere (Fig. 3, see above), are comparable to or larger than the climatological net air–sea heat fluxes^25^ (~10–200 W/m^2^) and mesoscale vertical fluxes (≲20 W/m^2^ over most of the ocean’s surface area; Supplementary Figure 3). Specifically, submesoscale vertical fluxes are more than five times larger than mesoscale vertical fluxes in 72% of ocean area in winter hemispheres (Fig. 3a, b vs Supplementary Figure 3a, b). The globally averaged submesoscale heat flux is 9 ± 3.5 W/m^2^, compared to a mesoscale heat flux of 1.5 ± 0.2 W/m^2^ (the global integrated values are ~3.0, 4.6, 2.0, and 2.9 PW for submesoscale flux in Fig. 3a–d, respectively, compared to only ~0.6, 0.6, 0.5, and 0.5 PW for mesoscale flux in Supplementary Figure 3a–d, respectively). These comparisons suggest a key role for submesoscale motions in controlling upper ocean vertical heat transport on a global scale. Mesoscale heat fluxes (Supplementary Figure 3) do not exhibit a seasonal cycle and are less systematically upward, which highlights a marked difference between mesoscale and submesoscale motions in modulating the near-surface heat budget. Moreover, this explicit quantification at submesoscales (Figs. 2–3) shows that the heat transport has a stronger temporal intermittency^24^, has larger amplitudes (~2–4 times larger), and exceeds 10 W/m^2^ over a greater proportion of the ocean (~3 times greater), as compared to studies that parameterize submesoscale motions^26^.

### Global air–sea heat flux modulated by submesoscale dynamics

**Fig. 4:**
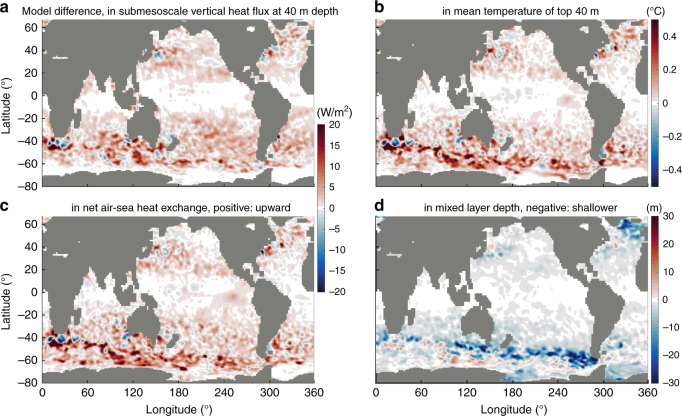
Sensitivity of the global heat budget to model resolution. Global maps of the annual-mean difference of **a** submesoscale vertical heat flux, **b** ocean surface temperature, **c** net air–sea heat flux, and **d** mixed layer depth between the 1/48° model and the 1/24° model (1/48° model minus 1/24° model). Values are spatially smoothed over 3° × 3° square boxes. The two models have identical setups except for the horizontal resolution. The model differences here are mainly caused by submesoscale at 10–20 km range. In most area of mid-latitudes, the 1/48° model exhibits upward heat transport at submesoscales larger than the 1/24° model, by ~4–10 W/m^2^ (**a**; about 14% larger, as in Supplementary Figure 5d). This results in a sea surface warming of ~0.06–0.3 °C (see **b**). This is ultimately compensated by a stronger upward ocean–atmosphere heat exchange (~4–10 W/m^2^, **c** vs **a**). The 1/48° model generally has a shallower mixed layer depth than the 1/24° model (see **d**). This is due to a stronger restratification in the 1/48° model (i.e., heat flux as in **a**) and is linked to most of the difference in sea surface temperature (see **b**). This is illustrated further in Supplementary Figure 4

These characteristics of the directly resolved submesoscale heat flux, in particular the large amplitudes and broad coverage (Figs. 2–3), suggest that its misrepresentation in coupled climate models could produce significant biases in the magnitude, the temporal variability, and the geographical location of ocean–atmosphere heat exchange. To test this, we compare two simulations with identical configurations except for the horizontal resolution (1/48° vs 1/24°). The nonlinear nature of turbulence (e.g., meso- or smaller scale fluctuations) could cause chaotic differences between two models due to the position of individual coherent structures. Therefore, we apply statistical averaging (annual mean, and 3° × 3° spatial mean) to our following analyses to compare statistical properties, although differences in coherent structure at larger scales, e.g., standing eddies in western boundary currents or the Antarctic Circumpolar Current (ACC), could still lead to differences between the two model runs (e.g., both positive and negative difference in these regions such as in Fig. 4). The 1/48° model exhibits a greater conversion from potential energy to kinetic energy (Supplementary Figure 5a), consistent with resolving submesoscale instabilities over a greater range of wavelengths, ~10–50 km, as compared to ~20–50 km for the 1/24° model (see “Methods”). Consequently, the global submesoscale field is more active in the 1/48° model (Supplementary Figure 5b–c). This produces stronger submesoscale upward heat fluxes in the upper ocean: 62% and 5% of the ocean area at 20°–60° mid-latitudes have fluxes that are more than 4 W/m^2^ and 10 W/m^2^ stronger, respectively (Fig. 4a and Supplementary Figure 5d; annual-mean here and for below). This acts to warm the sea surface (Fig. 4b): 64% and 8% of ocean area at 20°–60° mid-latitudes becomes more than 0.06 °C and 0.3 °C warmer, respectively. Again, these effects (Fig. 4) are mainly caused by submesoscale motions in the 10–20 km range. This result is further explained as follows: the stronger submesoscale upward heat flux in the 1/48° model (Fig. 4a and Supplementary Figure 5d), i.e., the stronger restratification flux, causes a shallower mixed layer in the 1/48° model (dashed and dotted curves in Supplementary Figure 4b; Fig. 4d). This process is equivalent to a stronger slumping of isopycnals^7,27^ (approximately temperature contours) in the 1/48° model (solid curves in Supplementary Figure 4b). This submesoscale-driven slumping of temperature contours explains the ocean surface warming (roughly within the range of wintertime mixed layer; Supplementary Figure 4b, b) and the deeper cooling (roughly below the wintertime mixed layer; Supplementary Figure 4b). This sea surface warming (Fig. 4b) results in a larger release of heat from the ocean to the atmosphere via intensified upward air–sea heat flux anomalies (Fig. 4c): 60% and 8% of the ocean area between 20° and 60° in both hemispheres show an intensification of the annual-average air–sea flux by more than 4 W/m^2^ and 10 W/m^2^, respectively. This increase in air–sea flux ultimately balances the stronger submesoscale upward heat flux (Fig. 4c vs 4a, see also explanation in “Methods”). This 4–10 W/m^2^ air–sea flux response is a significant fraction of the climatological, as well as our model’s, net air–sea heat flux, which is ≲20 W/m^2^ over broad regions of the ocean basins^25^.

## Discussion

Our numerical simulation of the global ocean has permitted an estimate of the vertical heat fluxes caused by submesoscale motions at ~10–50 km range (in terms of wavelengths, see “Methods”). This global ocean simulation is state of the art in terms of resolution; at the time of writing this manuscript, it remains impractical to go beyond a ~2 km resolution in a global model. We acknowledge that the heat flux contributions from submesoscale motions not resolved by this model (~0.1–10 km) could be significant and could quantitatively alter the values presented in this study. However, recent studies provide strong evidence that the total heat flux, including the full range of submesoscale motions, should remain upward (positive) and, if anything, are likely to be even larger than the values reported here. Indeed, a process study^12^ showed that submesoscale dynamics at the 0.1–10 km range leads to a significant restratification of the upper ocean (in particular through filamentary intensification) and therefore produce an upward heat flux. Other process studies^7,28–31^ suggested similar behavior. A more recent study^32^, using a non-hydrostatic model in a 200-km square domain, found that physical scales from 10 km down to 250 m lead to upward buoyancy fluxes and therefore upward heat fluxes, with a magnitude comparable to our estimate. Extrapolation of these localized or process-based results to the global ocean still needs to be examined.

An acknowledged limitation of this study is that the atmosphere and ocean are not fully coupled. In our global ocean simulation, modifications to sea surface temperature (SST) by submesoscale heat fluxes necessarily impact air–sea fluxes since the latter are parameterized using bulk formulae involving SST^33^. However, atmospheric variables, such as surface wind, air temperature, and humidity that also impact air–sea fluxes, are prescribed in our model, rather than being influenced by SST as they are in a fully coupled system. Thus, there is an additional uncertainty related to our calculated submesoscale and surface heat fluxes due to air–sea coupling. Submesoscale fluxes may modulate atmospheric dynamics that in turn lead to feedback on ocean dynamics. Indeed, multiple studies have pointed out the impact of eddy-driven SST anomalies on the stability of the overlying atmosphere^34^ and surface pressure anomalies^35^. These interactions may significantly influence wind stress^36–38^ and the location and strength of the atmospheric jet streams^39–41^, as well as mid-latitude storms^42^. This raises the possibility that SST changes reported in our study—most significant at mid-latitudes—may impact atmospheric storm-tracks, and therefore produce a feedback on the large-scale ocean circulation as well as on ocean submesoscale motions. This feedback may also include wind-front interactions^43^, wind-driven changes in mixed layer depth^44^ and ocean stratification,^45^ the change of wind energy input to the ocean^46^, and changes in air–sea turbulent fluxes^47^.

These results indicate that the oceanic submesoscale heat fluxes may impact ocean dynamics at both larger and smaller scales through the interaction with atmospheric dynamics. This impact may only be assessed and quantified through a global ocean–atmosphere-coupled model with a spatial resolution similar to the one considered in the present study. This simulation does not yet exist due to its computational expense.

We emphasize that critical regions for ocean heat uptake from the atmosphere^48^, e.g., the Southern Ocean and high-latitude North Atlantic, as well as regions that influence the atmospheric storm tracks, e.g., the Kuroshio Extension and Gulf Stream, are particularly affected by the strong submesoscale heat transport (~20–100 W/m^2^ in winter, Fig. 3). These regions suffer from uncertainty in the quantification of the local air–sea heat fluxes^49^. This can be partially attributed to the commonly neglected^26^ submesoscale heat transport, which may impact the partitioning of heat between the ocean and atmosphere (Fig. 4). Furthermore, the modification of air–sea heat fluxes due to submesoscale processes may influence the formation of deep, intermediate, and mode waters, which controls the downwelling and upwelling branches of the meridional overturning circulation^50^. Finally, this study has implications for the exchange of carbon and oxygen between the atmosphere, ocean surface, and ocean interior, which will be sensitive to near-surface submesoscale vertical fluxes. In particular, the Southern Ocean, where submesoscale activity is particularly strong (Fig. 3), accounts for up to 40% of global anthropogenic carbon uptake^51^. An important and likely computationally affordable next step is to explore the potential impact of oceanic submesoscale motions on atmospheric circulation and the feedback on air–sea flux and ocean state, using a high-resolution ocean model that is coupled to at least the atmospheric boundary layer. The impact of even smaller-scale oceanic motions than can be resolved in our study on the large-scale heat transport also needs to be explored, likely using a basin-scale ocean model.

## Methods

### Model description

We use a set of global, full-depth ocean and sea ice numerical simulations carried out using the Massachusetts Institute of Technology general circulation model (MITgcm) on a Latitude-Longitude polar Cap (LLC) grid^16^. The computation is enabled by NASA Advanced Supercomputing. The model output analyzed in this study is from the so-called LLC4320 simulation, which has a nominal horizontal grid spacing of 1/48° (0.75 km near Antarctica, 2.3 km at the Equator, and 1 km in the Arctic Ocean). We also use the LLC2160 simulation for the results presented in Fig. 4, which has a nominal horizontal grid spacing of 1/24°. Horizontal wavenumber spectra suggest^14^ that the effective resolution of LLC4320 and LLC2160 is about 10 km and 20 km, respectively. The 1/48° simulation spans 14 months from September 10, 2011 to November 15, 2012. The spin-up of this simulation is described in Appendix D and Table D2 of ref. ^16^; it progresses from a 1/6° global ocean state estimate generated by the Estimating the Circulation and Climate of the Ocean, Phase II (ECCO2) project^16^, to 1/12° and then 1/24° simulations. The 1/12°, 1/24°, and 1/48° simulations are forced with six-hourly surface atmospheric fields (10-m wind velocity, 2-m air temperature and humidity, downwelling long and shortwave radiation, and atmospheric pressure load) from the 0.14° European Centre for Medium-Range Weather Forecasting (ECMWF) atmospheric operational model analysis, starting in 2011. These three simulations also include tidal forcing for the 16 most significant components, applied as additional atmospheric pressure forcing. Vertical mixing is parameterized based on the critical value of Richardson number and is implemented using the K-Profile Parameterization (KPP) scheme^52^ that has been extensively used and evaluated in ocean modeling studies^53,54^. The 1/48° simulation has been evaluated using in situ observations in terms of the kinetic energy level at different wavelengths and frequencies^14,15^. This simulation’s air–sea fluxes have similar magnitudes as climatological fluxes. This is because our model’s global SST distributions are in realistic range and the air–sea fluxes in our model depend on SST and the prescribed reanalysis of atmospheric state such as near-surface air temperature, humidity, and wind speed according to the bulk formulae of ref. ^33^. This study focuses on submesoscales and its seasonality. The current integration of this simulation is able to reach the equilibrium state for submesoscale dynamics because of their short timescales. We are aware that it would take longer integration to reach the equilibrium state for the larger-scale ocean dynamics in the global ocean, which is beyond the capacity of the most powerful computers at the current time^50^. Note that the 1/48° simulation is integrated using primitive equations (i.e., with hydrostatic assumption). The horizontal grid spacing and the hydrostatic assumption indicate that the 1/48° simulation cannot adequately resolve symmetric instability. But to a large extent, we expect the 1/48° simulation to resolve some other crucial sources for generating submesoscale turbulence, such as the mixed layer instability^7,9,17,18,26^, wind-front interactions^43^, and strain-induced frontogenesis^3,28^.

### Relative vorticity ζ

Relative vorticity is defined as the rotational component of horizontal motions: *v*_*x*_ − *u*_*y*_, with *u* and *v* the zonal and meridional velocities and *x* and *y* the zonal and meridional coordinates; subscripts here imply partial differentiation. Here, ζ is a measure of the spin of a fluid parcel. A useful non-dimensional number characterizing the surface frontal structure is ζ/*f*, where *f* is the Coriolis parameter due to the Earth rotation. When this non-dimensional number (also called Rossby number, Supplementary Figure 1) is larger than 0.1, the vertical velocity associated with these frontal structures may be large^18,55,56^. About 10% of ocean area in the winter hemisphere (20°–60° latitude band) has a surface |Ro| ~0.4 (Supplementary Figure 1c). Root-mean-square Rossby number can reach ~0.4 during winter in basin-scale domains (Supplementary Figure 1d), suggesting that individual amplitudes of Rossby number can reach O(1). Smaller-scale motions, if resolved, should increase the Rossby number amplitudes.

### Definition and computation of submesoscale and mesoscale fields

We use the term submesoscale to designate a spatial scale just smaller than the mesoscale: a horizontal scale ≲0.5° in terms of longitude (~50 km at mid-latitudes), roughly below the first deformation radius in most of the global ocean^9,17–22^ (in terms of wavelengths, i.e., need to times a factor of *π*). The lower bound of submesoscales is often considered^3^ as O(0.1 km), but in our model is ~10 km, which is the effective resolution of our model^14^ since our numerical resolution is 1/48°, ~2 km at mid-latitudes (physical length scales really represented in the simulation are usually five times the numerical resolution). Therefore, submesoscale in this study refers to the range 1/10°–0.5° (~10–50 km). To focus on submesoscale processes, for all fields analyzed, we apply daily averaging to filter out most of the internal gravity waves^14^. The submesoscale component of ζ, denoted as ζ′, are the anomalies of the daily mean ζ from its 0.5° × 0.5° spatial mean (averaging over a 0.5° × 0.5° square box). Therefore, ζ′ is a spatial-temporal variable and approximately represents the component of ζ in the 1/48°–0.5° scale range (~2–50 km). Similarly, we can define the submesoscale component for other quantities such as temperature and vertical velocity. In our test, it yields essentially the same results when using 1° instead of 0.5° as the upper bound for defining submesoscales. The vertical heat flux (Figs. 2–4) is defined as *C*_p_*ρw*′*T*′, where *C*_p_ is the specific heat capacity, *ρ* is the density, *w*′ and *T*′ are the submesoscale components of vertical velocity and temperature, respectively. The spatial and temporal filters above have been similarly and widely used in other studies to deliver the submesoscale field^22,57,58^. The mesoscale component of a quantity is defined as the temporal anomalies of a given quantity from the annual average and has a spatial scale larger than 0.5° (averaging over a 0.5° × 0.5° square box)^22^.

### Sensitivity analysis shown in Fig. 4

We perform a sensitivity analysis by comparing two simulations with identical setups except for the horizontal resolution (1/48° vs 1/24°). The 1/48° model resolves submesoscale instabilities over slightly broader wavelengths than the 1/24° model^14^ (~10–50 km vs ~20–50 km). This powers a stronger global submesoscale field in the 1/48° model (Supplementary Figure 5a–c) and produce a stronger submesoscale upward heat transport in most area of mid-latitude upper ocean (Supplementary Figure 5d). This causes a warmer sea surface in the 1/48° model (Fig. 4b) and is ultimately balanced by a stronger upward ocean–atmosphere heat exchange (Fig. 4c). This response, to leading order, may be explained by a classic thermodynamic equation^59^ that captures the evolution of upper-ocean temperature *T*:

1 $${C}\;{{\rm{d}}T/{\rm{d}}{t}} = {{S}}-{{\lambda T}}.$$

Here *C* is the total heat capacity of the upper layer (the system we focus on), *S* is the heating source, and –*λT* is the negative feedback from air–sea heat flux, where *λ* can be approximated as a positive constant locally^59^. In the 1/48° model, *S* is larger due to a stronger incoming submesoscale heat flux from below (Fig. 4a). From Eq. (1), this causes a higher temperature *T* (Fig. 4b), and hence a larger upward air–sea heat exchange *λT* in the 1/48° model. The change in *λT* ultimately closely balance with the difference of *S* in the two model simulations (Fig. 4c vs 4a) such that the systems approach a quasi-steady state (d*T*/d*t* ~0 in an annual-mean sense). In this case for the upper 40 m ocean^59^, *λ* is ~15 Wm^2^/K and *C* is ~16 × 10^7^ Jm^2^/K. Therefore, the timescale *C*/*λ* is about a few months, which means that the air–sea flux (−*λT*) can respond to the forcing *S* by a timescale of months. This is captured in our simulations (see Supplementary Figure 4 for a further illustration). See the main text for the discussion of caveats.

### Code availability

The instructions and code for running the 1/24° and 1/48° simulations are available by the following websites:

http://wwwcvs.mitgcm.org/viewvc/MITgcm/MITgcm_contrib/llc_hires/llc_2160/

http://wwwcvs.mitgcm.org/viewvc/MITgcm/MITgcm_contrib/llc_hires/llc_4320/

### Data availability

The authors declare that the data supporting the findings of this study are available within the article and its Supplementary Information Files, or from the corresponding authors on request.

## Electronic supplementary material

Supplementary Information
