# Supplementary material for: Ocean submesoscales as a key component of the global heat budget
Source: Nat Commun. 2018 Feb 22;9:775. doi: 10.1038/s41467-018-02983-w (PMC5823912; doi:10.1038/s41467-018-02983-w)
Supplement: Supplementary file 1 — Supplementary Information [file 41467_2018_2983_MOESM1_ESM.pdf]

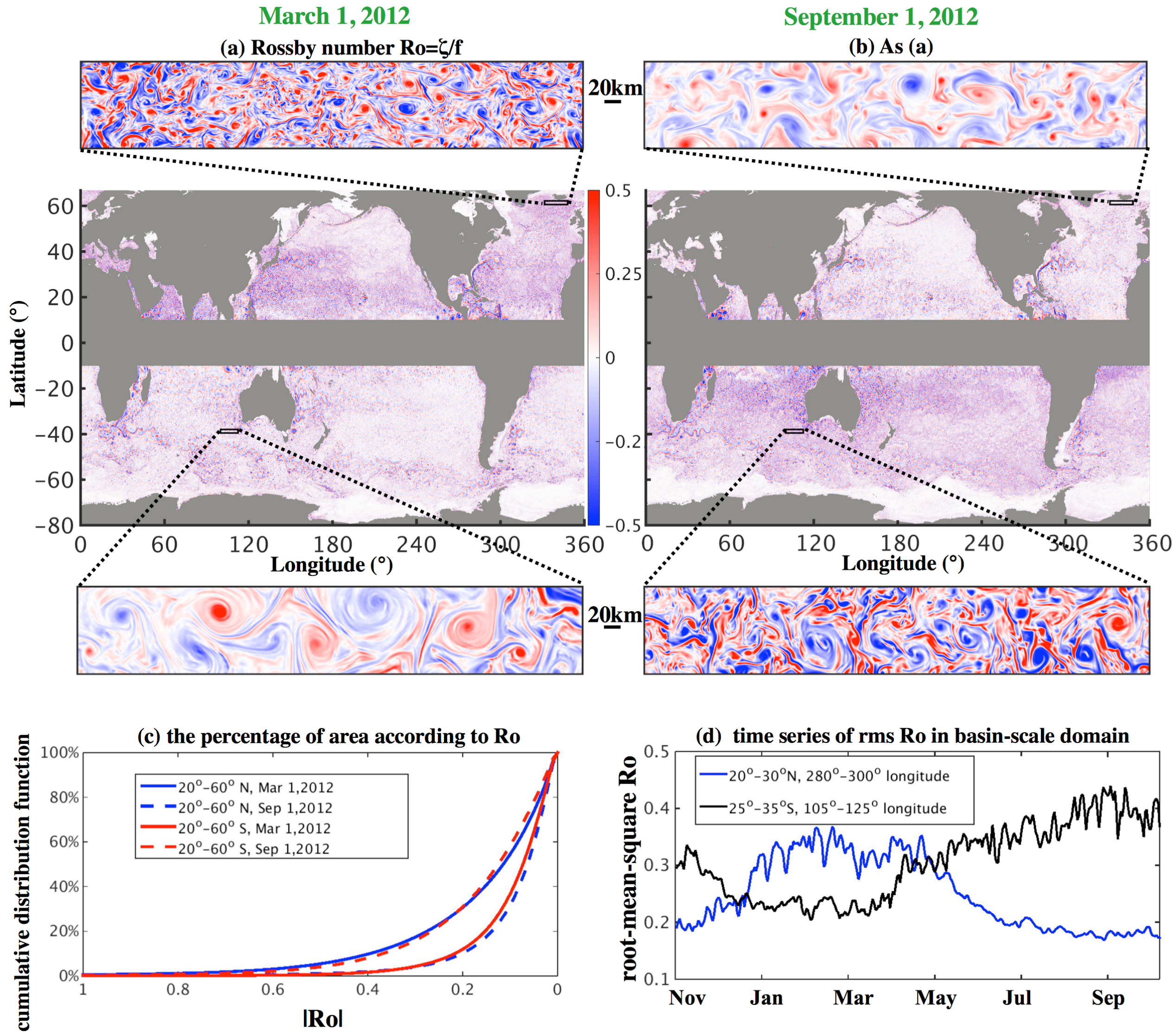

Supplementary Figure 1. (a)-(b) As Figures 1c-1d but for Rossby number Ro at the surface, defined as the relative vorticity ζ divided by the Coriolis frequency f. The equatorial band (10°S-10°N) is not shown due to the small values of f there. (c) Cumulative distribution function of surface |Ro| in each hemisphere (20º-60º latitude band). It shows that ~10% of ocean area in the winter hemisphere (20º-60º latitude band) has a |Ro| ~0.4 (blue solid curve, red dashed curve), which suggests a significant ageostrophic characteristic and hence large vertical velocity field in board ocean areas (Supplementary Figure 2). (d) Example time series of root-mean-square (rms) surface Ro in basin-scale domains. It shows that rms Ro in basin-scale domains can reach ~0.4 during winter, suggesting that individual/local amplitudes of Ro can reach O(1).

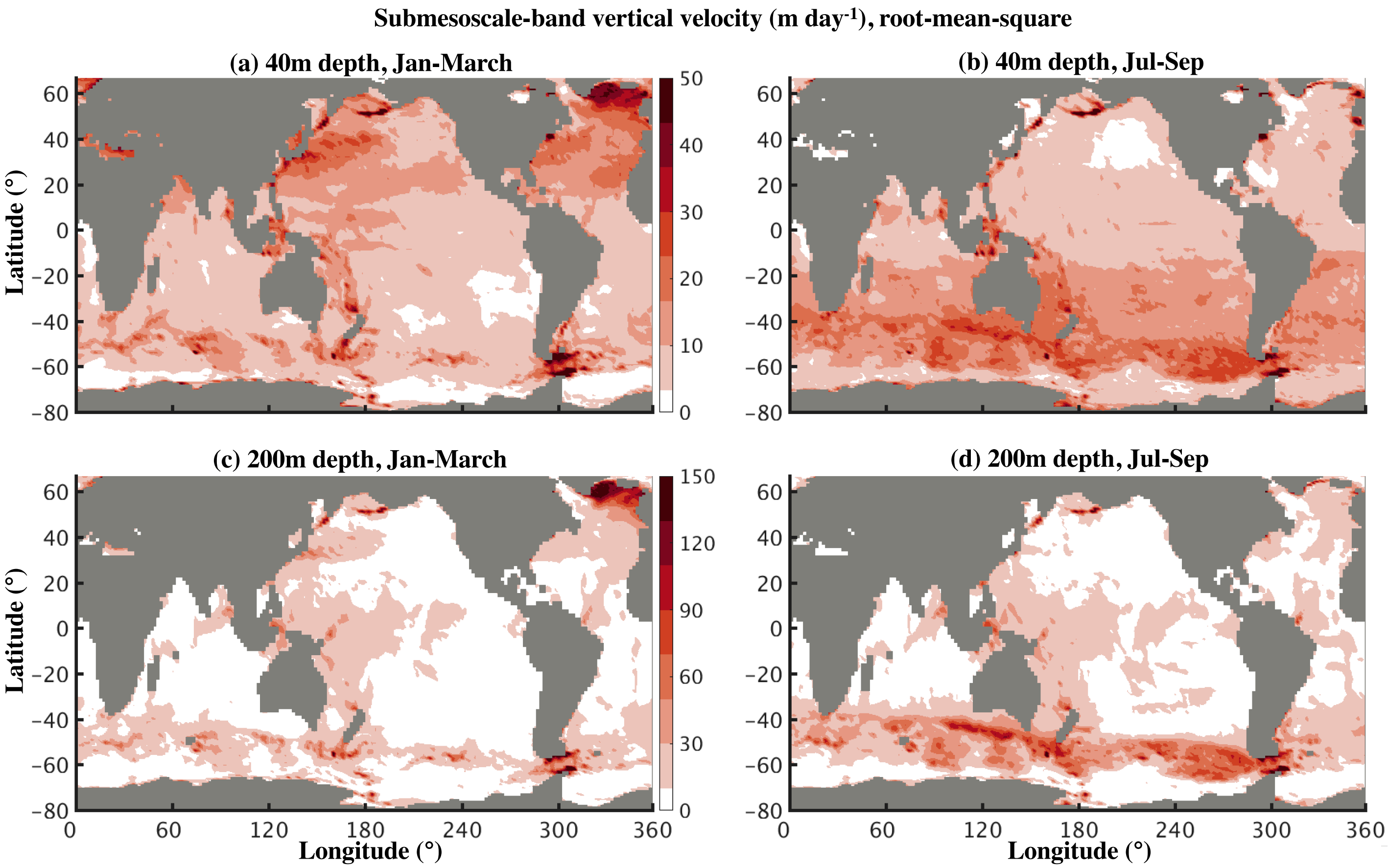

Supplementary Figure 2. (a)-(d) Global maps of the root-mean-square submesoscale-band vertical velocity (m s^{-1}. See method) at 40m and 200m depths during the winter or summer season (Jan-March mean, or Jul-Sep mean). The values are spatially smoothed over 3°×3° square boxes. These vertical velocities reach a large magnitude (≳10 m/day) in most area of wintertime mid-latitudes, suggesting a strong associated transport of heat (Figure 3).

2

3

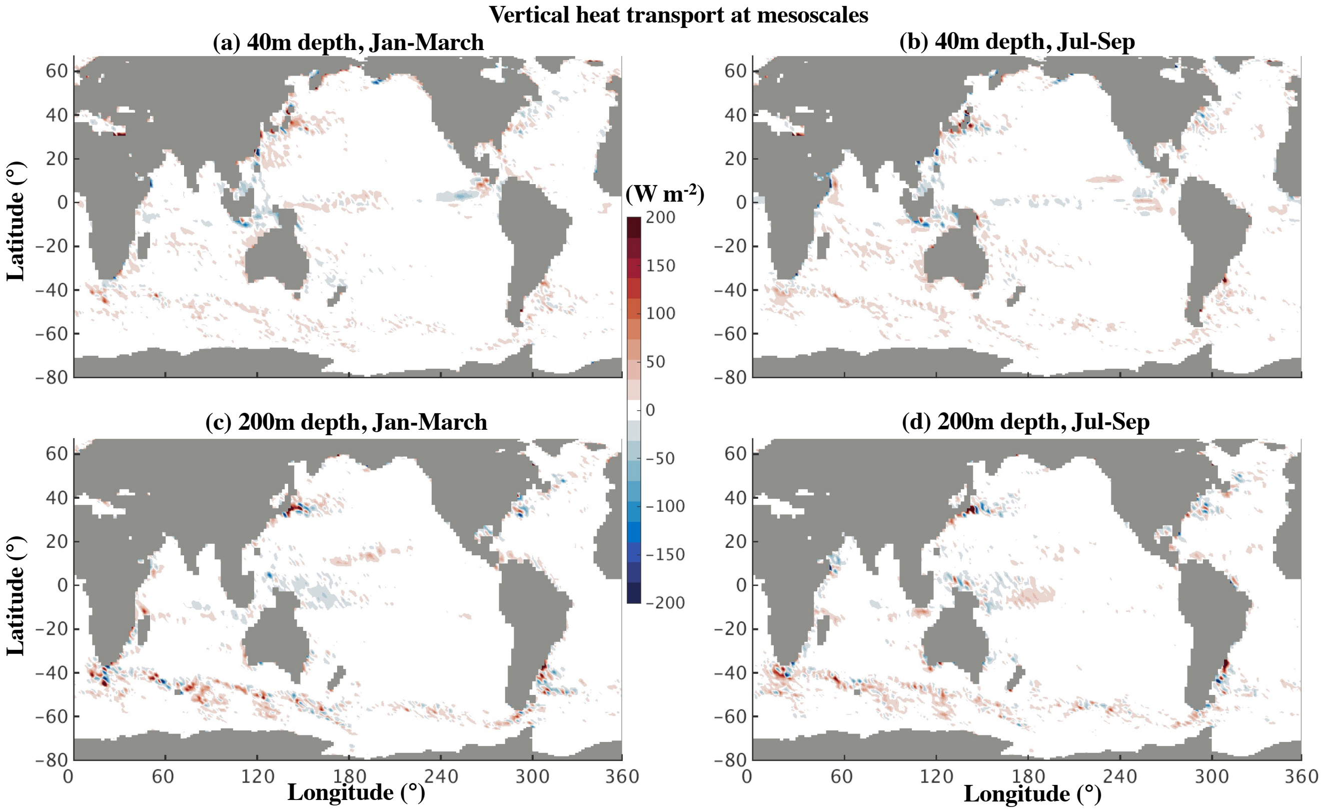

Supplementary Figure 3. (a)-(d) As Figure 3 but for the mesoscale band (see method). It has the same color scale as Figure 3. In contrast to submesoscales (Figure 3), upper-ocean vertical heat transport associated with mesoscale turbulence does not vary seasonally and has a much smaller spatial coverage. Unlike submesoscale heat flux, the mesoscale heat flux is not systematically upward (i.e. blue shows downward transport), making its seasonal averages much smaller than submesoscale flux (Figure 2; Figure 3 vs Supplementary Figure 3). Note that the submesoscale (0.1º-0.5º in term of longitude) and mesoscale (>0.5º) bands by this definition are not distinct bands of variability, and can have some overlap in dynamical properties, which is especially true at high latitudes. The mesoscale band here includes a wavelength and temporal range that has an upper bound of 1 year and a lower bound of ~50km (see method).

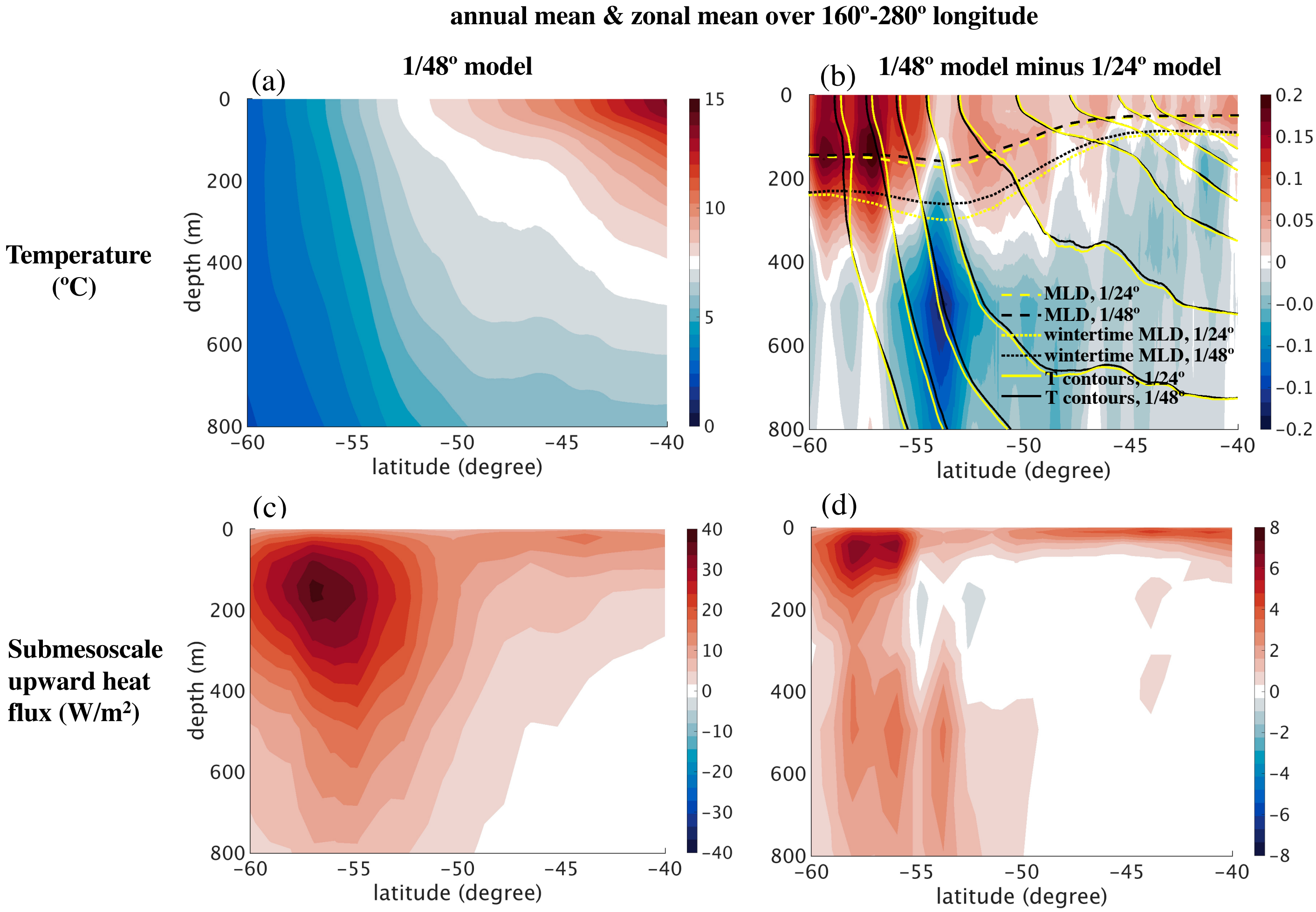

Supplementary Figure 4. A local example to further illustrate the results of Figure 4. All panels, except wintertime mixed layer depth in panel b, are annually averaged, and also zonally averaged over 160º-280º longitudes. All panels show a vertical section in the Southern Ocean (vertical section is very expensive to compute globally, and only the Southern Ocean is approximately zonally symmetric. So here we use a regional example in the Southern Ocean). The left panels (a,c) are for the 1/48º model and the right panels (b,d) show the difference between the 1/48º model and the 1/24º model. Panels (a, b) show temperature; panels (c, d) show submesoscale vertical heat flux (positive: upward). Including scales down to ~10km in the 1/48º model, as compared to ~20km in 1/24º model, causes a stronger submesoscale upward heat flux (panel d; Figure 4a), i.e., a stronger restratification flux. This submesoscale-driven stronger restratification causes a shallower mixed layer depth (MLD) in the 1/48º model (dashed and dotted curves in panel b; Figure 4d), which is equivalent to a stronger slumping of isopycnals (approximately temperature contours) in the 1/48º model (solid curves in panel b). This stronger slumping of temperature contours directly explains the ocean surface warming (roughly within the range of wintertime mixed layer; panel b, Figure 4b) and the deeper cooling (roughly below the wintertime mixed layer; panel b). Due to the weak stratification (the deep mixed layer) at 50º-60ºS, submesoscale heat flux has a peak at ~200m depth (panel c), then decays with depths.

7

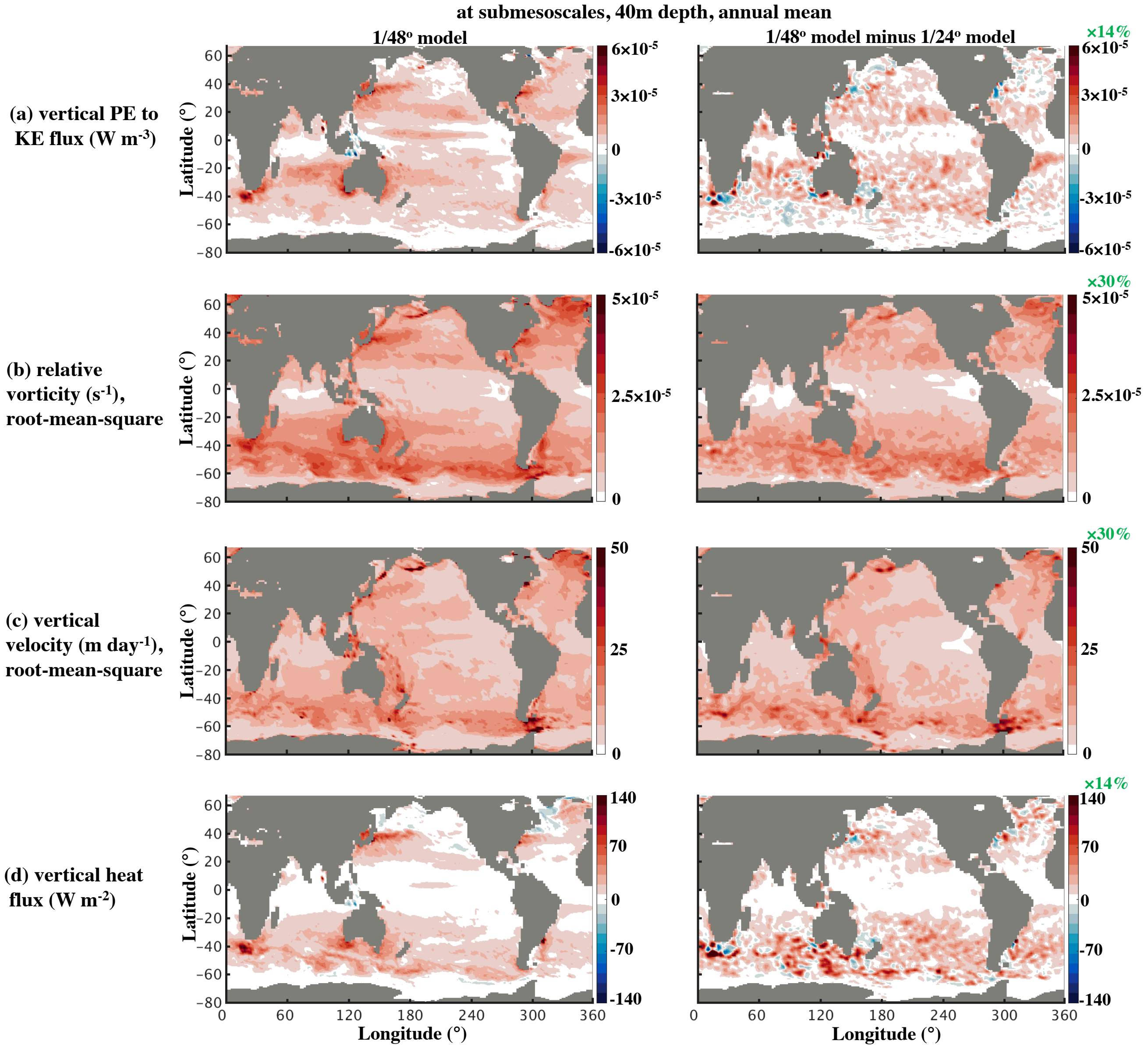

Supplementary Figure 5. (a)-(d) Global maps of annual-mean quantities at submesoscales at 40m depth (spatially smoothed over 3°×3° square boxes). The left panels are for 1/48° model and the right panels are the difference between 1/48° model and 1/24° model. Note that the color scales in the right panels are scaled by the percentage shown in green. The setups of two models are identical except for the horizontal resolution. Due to a higher resolution, the 1/48° model resolves more submesoscale instabilities that converts ~14% more potential energy (PE) to kinetic energy (KE) (panel a, $-w\rho g$). This powers a stronger submesoscale velocity field in the 1/48° model: ~30% stronger in terms of both relative vorticity and vertical velocity (panels b-c), and ~14% stronger in terms of upward heat transport (panel d). For panels a and d (right), the 1/48° model is stronger than 1/24° model as a whole, but can be weaker locally (blue). This is because these two depend on not only submesoscale strength, but also the tracer distribution (density and temperature) that is horizontally advected also by nonlinear mesoscale eddies.
